# A Case Report of Combined Type 2 Autoimmune Hepatitis and Antiphospholipid Syndrome Presenting With Intracranial Hemorrhage: Diagnostic and Therapeutic Dilemma

**DOI:** 10.7759/cureus.85337

**Published:** 2025-06-04

**Authors:** Shatabhisha Mandal, Chittaranjan Panda, Harpreet Singh, Nidhi Anand, Suresh Kumar

**Affiliations:** 1 Internal Medicine, Maulana Azad Medical College, New Delhi, IND; 2 Internal Medicine, IQ City Medical College and Hospital, Durgapur, IND

**Keywords:** antiphospholipid syndrome, autoimmune hepatitis, intracranial hemorrhage, platelet dysfunction, splenic artery embolization, thrombocytopenia

## Abstract

Background: Autoimmune hepatitis (AIH) is very frequently associated with many autoimmune diseases. Antiphospholipid syndrome (APS) is frequently associated with type 1 AIH but rarely seen in type 2 AIH patients. In this case, the association between type 2 AIH and APS is seen, along with complications, which made the management difficult.

Case presentation: This patient, who had a history of recurrent abortions, presented with complaints of recurrent epistaxis with severe thrombocytopenia. She was diagnosed with APS with concomitant type 2 AIH. After failing with conservative treatment, partial splenic artery embolization (PSAE) for recurrent thrombocytopenia was done, which led to improvement in platelet count and the resolution of recurrent epistaxis. But after a few days, the patient developed an intracranial hemorrhage (ICH) with a normal platelet count and coagulation profile. A platelet function study was done, and platelet dysfunction was found. Finally, after a prolonged period of admission, the patient developed sepsis and disseminated intravascular coagulation (DIC) and succumbed to death.

Conclusion: From this case, it may be kept in mind the possibility of the association of both types of AIH when dealing with a patient with APS. Unconventional methods such as partial splenic artery embolization can be useful in patients with recurrent thrombocytopenia. A platelet function study is a very important parameter to look for whenever a patient has bleeding with a normal coagulation profile and platelet count with isolated raised activated partial thromboplastin time (aPTT).

## Introduction

Autoimmune hepatitis (AIH) is a chronic inflammatory liver disease characterized by immune-mediated liver injury by molecular mimicry, often leading to chronic liver disease (CLD) and associated complications [1]. Autoantibodies, increased serum immunoglobulin G (IgG) levels, and interface hepatitis on histology are the characteristic features of this disease. It is more prevalent in genetically predisposed individuals, mostly associated with genes within chromosome 6 encoding human leukocyte antigen (HLA) class II DRB1 alleles. The disease develops after exposure to some triggering factors such as bacterial or viral infections, xenobiotics, chemical toxins, or drugs and is promoted by the decreased control of regulatory T-cells [2].

Though AIH is a rare disease, it can occur in patients all over the world. A study was done including data from three continents (Asia, Europe, and America) over a period of two years, which showed the incidence of 1.37 per 100,000 people per year and the prevalence of 17.44 per 100,000 people [3]. Another meta-analysis was done from 1970 to 2022 to see the prevalence and incidence of AIH, where the global incidence was seen to be 1.28 cases per 100,000 people per year and the prevalence was 15.65 cases per 100,000 people [4]. AIH can involve any age, any sex, and any ethnicity with a global distribution, but it is seen to be more prevalent in women than men (25%-30% of all patients) [2]. In the meta-analysis study done by Hahn et al., AIH was found to be more common in women than men, with an odds ratio of 3.10 [4]. The disease has a bimodal pattern of age distribution; that is, it is more prominent during childhood and the teenage group and again during the 4th-6th decades of life. The increased incidence of cases is now present even in older age after 65-70 years [2].

The clinical presentation of AIH varies greatly. Nearly 12%-35% of the patients are asymptomatic at diagnosis. The rest of the patients may present with recurrent fluctuating jaundice, signs of CLD, and, in advanced disease, signs of acute liver failure [2]. AIH is typically diagnosed using a simplified scoring system that evaluates clinical, serological, and histological findings. There are two main types of AIH based on serology: type 1 AIH, which is associated with antinuclear antibodies (ANA) and smooth muscle antibodies (SMA), and type 2 AIH, which is characterized by the presence of anti-liver-kidney microsomal (LKM) antibodies [1]. The overall incidence of type 1 AIH is one per 100,000 person-years, and the prevalence is 11 per 100,000 people. For type 2 AIH, the incidence and prevalence are 0.03 per 100,000 person-years and 0.43 per 100,000 people, respectively [4]. Overall, among all AIH patients, type 1 is present in 80% of the cases [2].

Antiphospholipid syndrome (APS) is an autoimmune disease that presents with recurrent abortions and the presence of venous or arterial thromboembolism. The pathophysiology is the presence of autoantibodies, that is, antiphospholipid antibodies. The diagnosis is made using the revised Sapporo criteria, which includes clinical and laboratory criteria. Grossly, the clinical criteria includes recurrent abortions and/or the presence of venous or arterial thrombosis, and the laboratory criteria includes the presence of lupus anticoagulant and/or medium or high titers of anticardiolipin antibody and/or anti-beta-2 glycoprotein detected on two or more occasions at least 12 weeks apart. There are some supportive findings that are not included in the criteria, which are the presence of thrombocytopenia, nephropathy, cognitive impairment, valvular heart disease, etc. [5].

The prevalence of APS is nearly 50 per 100,000 population, and the annual incidence is 2.1 per 100,000 person-years. This disease also has a female predominance, especially during reproductive age (55%-67%), after which the male-to-female ratio becomes nearly equal. Though the incidence of APS increases in women in the age group of 35-39 years, the mean age of diagnosis is around 50 years of age. The most common presentation of this disease is recurrent abortions, placental insufficiency, and increased pregnancy morbidity. Among thrombotic events, venous thromboembolism is more common than arterial thromboembolism. In arterial involvement, patients present with increased cardiovascular events, ischemic strokes, renal artery thrombosis, and ischemic limb [5].

Autoimmune hepatitis very commonly overlaps with other various autoimmune diseases such as hypothyroid or hyperthyroid disorders (10%-20%), rheumatoid arthritis (40%), celiac disease (3.5%), inflammatory bowel disease, type 1 diabetes mellitus, psoriasis, systemic lupus erythematosus, alopecia, and type 1 autoimmune polyendocrinopathy syndrome (10%-18%) [2,6]. Type 1 AIH is frequently associated with APS, but the exact prevalence of APS in type 1 AIH is yet to be determined. In a case series done by Branger et al., it was found that the frequency of antiphospholipid antibodies was 70.8% in AIH patients, with 16.6% of patients having well-defined APS [7]. In another study by Liaskos et al., anticardiolipin antibodies were found in 39% of type 1 AIH patients [8]. Few case reports were found where APS and AIH were coexistent in the patient, along with some other disease-related complications, but the type of AIH was not mentioned in these articles [9,10]. No case report was found on the co-occurrence of type 2 AIH with APS.

This case report indicates the rare association of type 2 AIH with APS. It also highlights the diagnostic and therapeutic challenges in managing overlapping autoimmune syndromes presenting with complications.

## Case presentation

A 30-year-old woman presented with epistaxis for the past four days, with a history of recurrent episodes of epistaxis over the past five years. Each episode involved a few drops of blood that stopped spontaneously within minutes. She also had a history of headaches for eight years, during which she was diagnosed with a temporal lobe ischemic stroke at the All India Institutes of Medical Sciences (AIIMS), Delhi. Additionally, she had a history of two abortions at 16 and 20 weeks of gestation.

Initially, the patient visited the ENT department where evaluation ruled out local causes of the nasal bleeding, and the patient was referred to the medicine department for underlying pancytopenia. On examination, she had pallor, and there were blood clots in the nasal mucosa and splenomegaly on abdominal examination; other findings were normal. Initial investigations showed pancytopenia (Table 1).

**Table 1:** Routine blood investigations on admission and progress during hospital stay PSAE, partial splenic artery embolization; SI, International System of Units

Routine investigations (SI units)	Day 1	Day 36 (post-PSAE)	Normal range
Hemoglobin (g/L)	77	90	120-150
Total leucocyte counts (×10^9^ cells/m^3^)	3	8	4-11
Platelets (×10^7^ cells/m^3^)	4.6	8.2	15-45
Alanine transaminase (U/L)	29	41	<35
Aspartate transaminase (U/L)	32	82	14-36
Alkaline phosphatase (U/L)	261	146	38-126
Total bilirubin (µmol/L)	30.8	53.0	3.4-22.2
Urea (mmol/L)	4	6.5	2-6.5
Creatinine (µmol/L)	53.05	53.05	53.05-106.1
Sodium (mmol/L)	141	136	137-145
Potassium (mmol/L)	3.6	4.2	3.5-5.1
International normalized ratio	1.1	1.0	0.8-1.2

On further investigation, abdominal ultrasound suggested chronic liver disease (CLD) with portal hypertension and splenomegaly (Table 2), which was confirmed later by FibroScan of the liver (Table 3), which showed liver stiffness or elasticity of 26.9 kPa, suggesting liver cirrhosis. Other causes of CLD were excluded, and further evaluation for autoimmune liver disease was initiated. Elevated serum immunoglobulin G (IgG) levels and positive anti-LKM antibodies led to a provisional diagnosis of type 2 autoimmune hepatitis (Table 3). A liver biopsy was planned for confirmation.

**Table 2:** Radiological investigations and their findings IHBRD, intrahepatic biliary radicle dilatation; CBD, common bile duct; IVC, inferior vena cava

Investigation	Result
Ultrasound of the whole abdomen	Liver: 10.9 cm, no IHBRD, increased echogenicity, and irregular surface. Spleen: 13.6 cm and normal echogenicity. Portal vein: 15 mm. Gallbladder (GB): distended and echo-free. CBD: not dilated
Splenic and portal axis Doppler	Portal vein: 14.4 mm with hepatopetal flow. IVC: 2.7 cm. Hepatic vein: 9.7 mm
Magnetic resonance cholangiopancreatography (MRCP)	Left lobe and caudate lobe hypertrophy in the liver with gross splenomegaly (18.4 cm) with prominent splenoportal axis, perisplenic collaterals, and reactive GB wall thickening with pericholecystic fluid
Upper gastrointestinal endoscopy	Portal gastropathy present

**Table 3:** Special blood investigations for further diagnosis HIV, human immunodeficiency virus; HBsAg, hepatitis B surface antigen; Anti-HCV Ab, anti-hepatitis C virus antibody; IgG, immunoglobulin G; IgM, immunoglobulin M; CAP, controlled attenuation parameter; E, elastography; SI, International System of Units

Special investigations (SI units)	Patients value	Normal range
Vitamin B12 (pmol/L)	478.7	145.7-569.1
Folic acid (nmol/L)	36.2	7.0-46.4
Iron (µmol/L)	10	8.8-32.4
Total iron-binding capacity (µmol/L)	27.6	47.4-89.0
Serum ferritin (pmol/L)	48.8	49.2-619.8
Reticulocyte count	1.5%	0.5%-2.5%
Indirect Coombs test	Negative	Negative
Direct Coombs test	Negative	Negative
D-dimer (µg/L)	>Max	<250
Activated partial thromboplastin time (seconds)	90	21-35
Uric acid (µmol/L)	184.2	148.4-367.6
Lactate dehydrogenase (U/L)	233	120-246
HIV I and II	Non-reactive	Non-reactive
HBsAg	Non-reactive	Non-reactive
Anti-HCV Ab	Non-reactive	Non-reactive
Serum IgG (g/L)	22	<17
Antinuclear antibody (indirect immunofluorescence)	Negative	Negative
Antineutrophil cytoplasmic antibody (U/mL)	Negative	Negative
Anti-liver-kidney microsomal antibody (U/mL)	17	<15
Anti-cardiolipin IgM antibody (IU/mL)	12.63	<6.8
Anti-beta-2 glycoprotein IgM antibody (IU/mL)	21	<13.6
FibroScan of the liver	CAP=193; dB/m E=26.9 kPa	CAP<238; dB/m E<6 kPa

Given her history of ischemic stroke and recurrent abortions, antiphospholipid antibodies were sent, which came back positive (Table 3); thus, the diagnosis of APS was made. The medical management for thrombocytopenia was continued, but there was no significant improvement even after multiple platelet transfusions. The patient was developing progressive jaundice, and magnetic resonance cholangiopancreatography (MRCP) was planned. On MRCP, the patient had a splenomegaly of 18.4 cm (Table 2).

Consultation taken from the gastroenterology department and partial splenic artery embolization (PSAE) were suggested for a reactive spleen causing recurrent thrombocytopenia. Percutaneous partial splenic artery embolization using intravascular detachable coils and polyvinyl alcohol particles was performed by the cardiology department, along with the help of the gastroenterology department. Post-procedure, her platelet counts improved, her epistaxis resolved, and she was discharged with a plan to do a liver biopsy on follow-up. Due to her recent bleeding history, anticoagulation therapy was deferred.

**Figure 1:**
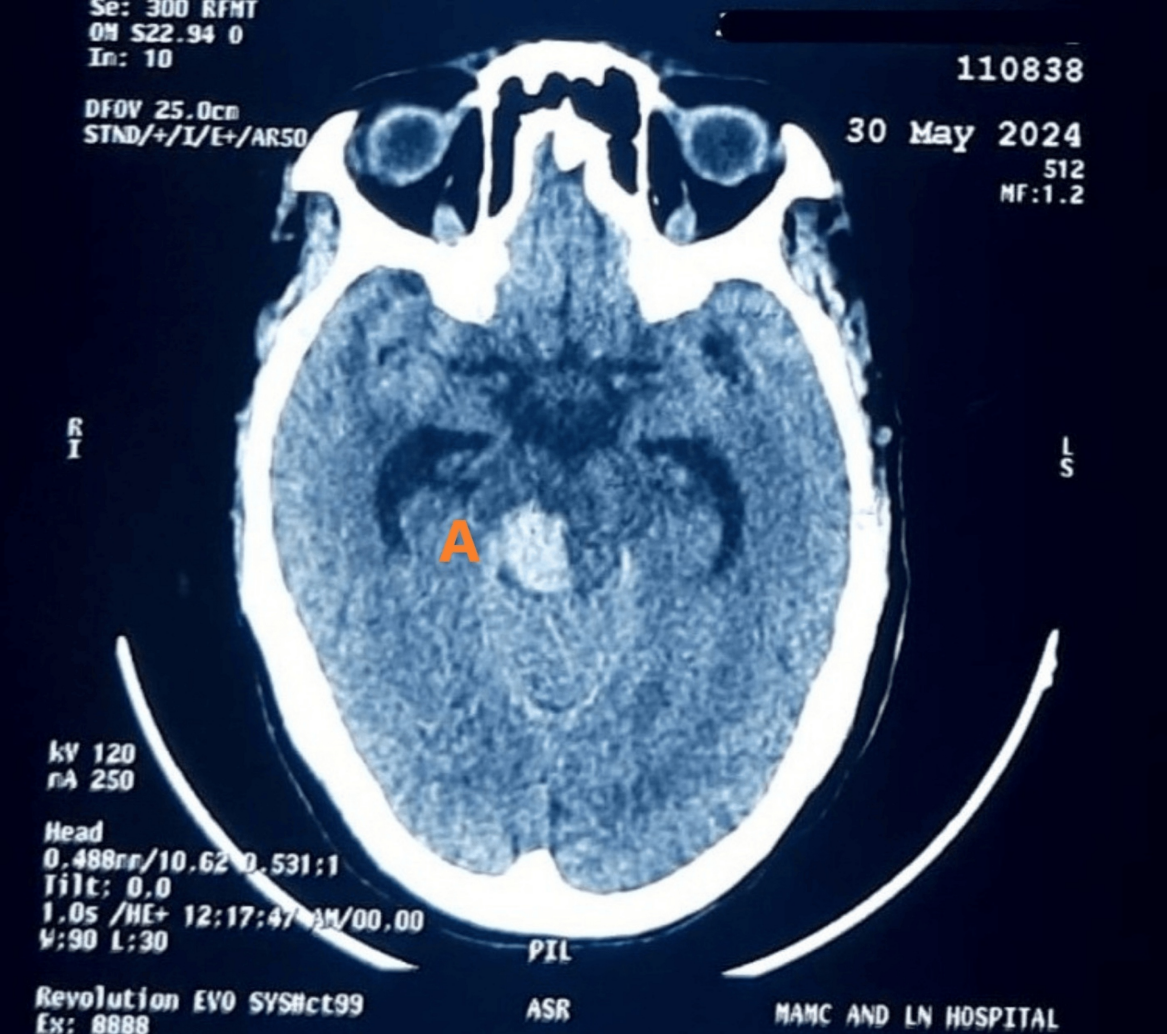
NCCT of the head following altered sensorium showing pontine hemorrhage on the right side (labelled as A) NCCT: non-contrast computed tomography

Before the liver biopsy could be conducted, she was readmitted with perrectal bleeding. On examination, hemorrhoids were seen and were managed medically. Upper GI endoscopy also suggested portal gastropathy (Table 2), and the patient was started on beta-blockers. After one day following improvement, the patient developed a sudden-onset altered sensorium. Clinical examination pointed toward stroke, and non-contrast CT of the head confirmed a pontine hemorrhage (Figure 1).

She was managed conservatively after neurology review but did not improve. At that time, her blood pressure, platelet count, and prothrombin time (PT)/international normalized ratio (INR) were normal, but activated partial thromboplastin time (aPTT) came out to be prolonged, following which a platelet function study was done (Table 4).

**Table 4:** Platelet function study of the patient Max aggregation: the highest point of platelet clumping in response to the agonist. Time to max: Time to reach the peak response ADP: adenosine diphosphate

Parameters	Max aggregation (%)		Time to max (seconds)	
	Patient’s value	Normal	Patient’s value	Normal
ADP	82.2	70-100	450	200-300
Collagen	7.6	70-100	599	300-500
Epinephrine	93.6	60-90	591	<500
Ristocetin	90.7	70-100	263	200-400

In this study, there was reduced agonist-induced response to collagen and adenosine diphosphate (ADP) suggestive of platelet aggregation disorder. Normal aggregation response was seen to epinephrine and ristocetin. Unfortunately, gradually, the patient developed aspiration pneumonitis, sepsis, and disseminated intravascular coagulation (DIC) and eventually succumbed to these complications.

## Discussion

Autoimmune hepatitis has a significant correlation with the presence of antiphospholipid antibodies [11]. Though there is very little information on why such a correlation is present, there are hypotheses such as the disruption of liver cell membranes leading to the chronic stimulation of neoantigens and the induction of antibody formation [8] or immunological cross-reactivity between nonpathological autoantibodies [12].

In this case, the patient had presented with thrombocytopenia, due to which she had symptoms of epistaxis on presentation. An increase in portal vein pressure can cause an increase in splenic sequestration of platelets in CLD patients, and along with that, splenomegaly negatively correlates with platelet count [13]. Morbidity varies quite greatly from 6% to 78% in CLD patients with thrombocytopenia [14]. Conservative management, along with platelet transfusion, was done in this case in view of recurrent thrombocytopenia, but it did not improve. Rather, it raised the chances of increased platelet activation and thrombin/antithrombin complex generation in a patient with concurrent APS [15]. Splenic artery embolism had a significant benefit in some cases of recurrent thrombocytopenia. Though total splenic artery embolization had numerous complications, in comparison to that, partial splenic artery embolization had more benefits and fewer complications [16]. Recent studies showed significant correction of thrombocytopenia in patients with CLD with hypersplenism and also improvement in recurrent bleeding from esophageal varices post-PSAE [17,18]. In this case also, a significant improvement in platelet count was seen after partial splenic artery embolization.

Initially, the patient improved significantly, but then, she developed an intracerebral hemorrhage (ICH) and started deteriorating clinically. As the patient had a normal platelet count by then and, in the coagulation study, only aPTT was raised, a platelet function study was done. In this patient, the platelet function test showed a platelet aggregation defect, and normal aggregation response to epinephrine and ristocetin suggested a normal coagulation pathway and von Willebrand factor function. In cirrhotic patients, there is a defect in platelet glycoprotein 1b due to elevation in circulating von Willebrand factor. There is also an acquired glycoprotein VI signalling defect in cirrhosis, which ultimately leads to platelet dysfunction [15]. In this case, cirrhosis may have caused a platelet function defect, along with APS and lupus anticoagulant antibodies, which also have a tendency to cross-react with beta-2 glycoprotein 1 leading to increased chances of bleeding episodes. All of these diseases can cause prolonged activated partial prothrombin time [19]. In this patient, all of these factors may have contributed to the pontine hemorrhage and caused deterioration.

As the patient had a diagnosis of APS both clinically and serologically, anticoagulation treatment was planned, but as she had epistaxis at the initial stage, it was not given. After improvement, she again developed perrectal bleeding, followed by intracerebral hemorrhage, so anticoagulation therapy was again deferred. A liver biopsy was planned for the confirmation and better management of autoimmune hepatitis, but initially, thrombocytopenia and procedures such as PSAE and, later, the deterioration of the patient by sepsis and multiorgan dysfunction, along with coagulation defects, prevented the biopsy procedure.

## Conclusions

This case was an overlap of two autoimmune diseases, type 2 AIH and APS. The presence of antiphospholipid antibodies in type 1 AIH is not uncommon, as discussed previously, but in the case of type 2 AIH, no proper study has been found. One challenging aspect of managing such complex cases is addressing complications such as severe thrombocytopenia due to splenomegaly in CLD patients. In this particular case, splenic artery embolization effectively increased platelet counts and resolved the recurrent epistaxis. However, the patient later developed an intracranial hemorrhage (ICH), for which coagulation studies did not provide much explanation, and a platelet function study finally revealed platelet dysfunction. During the management of the patient, anticoagulation could not be given to the patient due to bleeding complications. Managing intracranial hemorrhage was also difficult in this patient.

From this case, it may be kept in mind the possibility of the association of both types of AIH when dealing with a patient with APS. Unconventional methods, such as partial splenic artery embolization, in the management of recurrent thrombocytopenia might help in the improvement of patients, especially in hypersplenism. In case of bleeding from an unknown cause, platelet function studies can be considered when the patient has raised aPTT with other normal parameters. So, this case gives a wide range of prospects to think about while dealing with such complex patients.
